# Supplementary material for: From sequence to enzyme mechanism using multi-label machine learning
Source: BMC Bioinformatics. 2014 May 19;15:150. doi: 10.1186/1471-2105-15-150 (PMC4229970; doi:10.1186/1471-2105-15-150)
Supplement: Additional file 2 — Java code of ml2db. Additional file ml2db_code.tar.gz contains the Java source code to run the multi-label machine learning experiments and save the results to database. The code’s Javadoc is included. [file 1471-2105-15-150-S2.zip › additional file 2/ml2db/ml2db/doc/mulan/evaluation/loss/package-summary.html]

mulan.evaluation.loss


JavaScript is disabled on your browser.


- Overview
- Package
- Class
- Use
- Tree
- Deprecated
- Index
- Help

- Prev Package
- Next Package

- Frames
- No Frames

- All Classes

# Package mulan.evaluation.loss

- Interface Summary

  | Interface | Description |
  | --- | --- |
  |  |  |
  | --- | --- |
  | BipartitionLossFunction | Interfance for bipartition loss functions |
  | MultiLabelLossFunction | Interfance for loss functions |
  | RankingLossFunction | Interfance for ranking loss functions |
- Class Summary

  | Class | Description |
  | --- | --- |
  |  |  |
  | --- | --- |
  | BipartitionLossFunctionBase | Base class for bipartition loss functions |
  | ErrorSetSize | Implementation of the ErrorSetSize loss function, which computes the size of the error set. |
  | HammingLoss | Implementation of the hamming loss function. |
  | HierarchicalLoss | Implementation of the hierarchical loss function. |
  | IsError | Implementation of the IsError loss function, which is simply the indicator of whether the induced ranking is perfect or not. |
  | OneError | Implementation of the one-error loss function. |
  | OneMinusAveragePrecision | Implementation of the average precision as loss function. |
  | RankingLoss | Implementation of the "ranking loss" ranking loss function. |
  | RankingLossFunctionBase | Base class for ranking loss functions |

- Overview
- Package
- Class
- Use
- Tree
- Deprecated
- Index
- Help

- Prev Package
- Next Package

- Frames
- No Frames

- All Classes
